# Effects on muscle tissue remodeling and lipid metabolism in muscle tissue from adult patients with polymyositis or dermatomyositis treated with immunosuppressive agents

**DOI:** 10.1186/s13075-016-1033-y

**Published:** 2016-06-10

**Authors:** Ingela Loell, Joan Raouf, Yi-Wen Chen, Rongye Shi, Inger Nennesmo, Helene Alexanderson, Maryam Dastmalchi, Kanneboyina Nagaraju, Marina Korotkova, Ingrid E. Lundberg

**Affiliations:** Karolinska Institutet, Department of Medicine, Rheumatology Unit, Karolinska University Hospital Solna, Stockholm, Sweden; Childrens National Medical Center, Research Center for Genetic Medicine, Washington, DC USA; Center for Human Immunology, Autoimmunity and Inflammation, National Heart/Lung and Blood Institute, National Institutes of Health, Bethesda, Maryland USA; Karolinska University Hospital Huddinge, Institution for Laboratory Medicine (LABMED), Stockholm, Sweden; Karolinska Institutet, Department of NVS, Division of Physical Therapy and Karolinska University Hospital Solna, Physical Therapy Clinic, Stockholm, Sweden

**Keywords:** Glucocorticoids, Treatment, Muscle biopsies, Polymyositis, Dermatomyositis, Gene expression profiling

## Abstract

**Background:**

Polymyositis (PM) and dermatomyositis (DM) are autoimmune muscle diseases, conventionally treated with high doses of glucocorticoids in combination with immunosuppressive drugs. Treatment is often dissatisfying, with persisting muscle impairment. We aimed to investigate molecular mechanisms that might contribute to the persisting muscle impairment despite immunosuppressive treatment in adult patients with PM or DM using gene expression profiling of repeated muscle biopsies.

**Methods:**

Paired skeletal muscle biopsies from six newly diagnosed adult patients with DM or PM taken before and after conventional immunosuppressive treatment were examined by gene expression microarray analysis. Selected genes that displayed changes in expression were analyzed by Western blot. Muscle biopsy sections were evaluated for inflammation, T lymphocytes (CD3), macrophages (CD68), major histocompatibility complex (MHC) class I expression and fiber type composition.

**Results:**

After treatment, genes related to immune response and inflammation, including inflammasome pathways and interferon, were downregulated. This was confirmed at the protein level for AIM-2 and caspase-1 in the inflammasome pathway. Changes in genes involved in muscle tissue remodeling suggested a negative effect on muscle regeneration and growth. Gene markers for fast type II fibers were upregulated and fiber composition was switched towards type II fibers in response to treatment. The expression of genes involved in lipid metabolism was altered, suggesting a potential lipotoxic effect on muscles of the immunosuppressive treatment.

**Conclusion:**

The anti-inflammatory effect of immunosuppressive treatment was combined with negative effects on genes involved in muscle tissue remodeling and lipid metabolism, suggesting a negative effect on recovery of muscle performance which may contribute to persisting muscle impairment in adult patients with DM and PM.

## Background

Polymyositis (PM) and dermatomyositis (DM) are chronic, idiopathic inflammatory myopathies (IIM) characterized by proximal muscle weakness. Muscle biopsies reveal signs of inflammation including infiltrating T cells, macrophages, cytokines (interleukin (IL)-1) interferons (IFNs)) and upregulated major histocompatibility complex (MHC) class I expression in the fibers as well as regenerating and degenerating fibers [1, 2]. Treatment is based on high doses of glucocorticoids (GC) often combined with additional immunosuppressive drugs. The effectiveness of GC in patients with PM or DM varies between individuals, but is often disappointing and few recover former muscle performance [3–5]. In addition, side effects such as osteoporosis, hypertension, insulin resistance and steroid myopathy are common [6].

GC interact with the glucocorticoid receptor (GR) and form a complex that is translocated into the cell nucleus where it regulates target gene actions through transrepression or transactivation mechanisms [7–9]. It is assumed that the immunosuppressive and anti-inflammatory effects of GC are mediated through transrepression, downregulating the expression of pro-inflammatory cytokines such as IL-1, tumor necrosis factor (TNF) and IFNγ [9]. On the other hand, transactivation through GC response elements (GREs) controls genes that mediate metabolic side effects of GC and enhances the expression of anti-inflammatory genes such as IL-10, IKB and annexin-1 [9]. The limited effects of conventional immunosuppressive treatment, including high doses of GC, on muscle performance in patients with PM and DM is well recognized, but the underlying molecular mechanisms of the limited effects have not been completely elucidated. Persisting upregulation of certain inflammatory pathways such as infiltrating T cells, MHC-I, several pro-inflammatory cytokines [10–12], prostaglandin E_2_ (PGE_2_) [13] and leukotriene B_4_ (LTB_4_) pathways [14] in muscle tissue might partly explain the sustained weakness in patients despite treatment. Other molecular mechanisms affected by treatment may also influence muscle performance. This emphasizes the need for a better understanding of the molecular response in the target organ (muscle) in order to identify new therapeutic targets and abolish the persistent muscle weakness.

In this study, we aimed to investigate molecular events that might contribute to persisting compromised muscle function despite immunosuppressive treatment in adult patients with PM or DM. Thus, we investigated muscle biopsies taken before and after conventional immunosuppressive treatment using gene expression profiling combined with analysis of selected proteins at the protein level.

## Methods

### Patients and muscle biopsies

From an observational study, six untreated adult patients of Caucasian origin diagnosed with probable or definite DM or PM [15] were all subject to follow-up biopsies for the study. Disease duration was defined from the first reported symptom related to disease to time of the first muscle biopsy. Clinical data including support for diagnosis are presented in Table 1. All adult patients were initially treated with oral prednisolone (0.75 mg/kg/day) in combination with an additional immunosuppressive drug (methotrexate or azathioprine) as decided by the treating physician. Muscle tissue biopsies were taken from *m. vastus lateralis*; a repeated biopsy was taken after 9 months (range 8–15 months) of conventional immunosuppressive treatment [16]. None of the patients exercised at the time of the first biopsy, but all were instructed to a 5-days-a-week home exercise program after introduction of glucocorticoids. Patients one, two, four, and five exercised regularly with the home exercise program or more intensive gym training 1–2 times a week during the study period. The regional ethics committee in Stockholm granted approval (approval number: 2005/792-31/4) and all participants gave informed consent to participate in the study.

**Table 1:** Clinical data on the patients at the time of biopsies

Patient ID	Diagnosis	Age (years)	Gender	Disease duration (months)	Cumulative cortisone (mg)	Imuunosuppressive treatment at second biopsy	Autoantibodies	Support for diagnosis	MMT-8 (%)		s-CK (μcat/L)		HAQ (0.0–3.0)		FI-2 (%)	
									B	A	B	A	B	A	B	A
1	DM	40	M	3	14450.0	Pred, AZA	MDA5, SSA, Ro60	MW, S, LD	87.5	87.5	1.3	1.1	1.13	0.75	21.7	27.4
2	PM	73	F	12	6222.5	Pred, MTX	Neg	MW, CK, MB	85.0	85.0	10.2	1.5	0.5	0.88	18.3	19.1
3	DM	69	F	2	10017.5	Pred, MTX	ANA, TIF1γ	MW, CK, S, EMG	81.4	91.3	29.3	1.0	0.63	0	0	11.2
4	DM	63	M	0.5	7330.0	Pred, AZA	Neg	MW, CK, S, EMG	78.8	90.0	48.2	1.0	0,38	0	86.2	100.0
5	DM	45	F	16	6842.5	Pred, MTX	ANA, Mi-2	MW, CK, MB, S, EMG	98.8	87.5	43.0	2.1	1.5	0.88	17.3	25.3
6	PM	71	F	12	8415.0	Pred, AZA	Neg	MW, CK, MB	75.7	100	9.0	3.9	1.25	1.88	NA	17

*A* after treatment, *ANA* antinuclear antibodies, *AZA* azathioprine, *B* before treatment, *CK* creatine kinase (reference interval, male: 3.3 μkat/L, female: 2.5 μkat/L), *DM* dermatomyositis, *EMG* positive for electromyography, *F* female, *FI-2* Functional Index-2 (0–100 %; impairment in performing repetitions, respective no impairment), *HAQ* Health Assessment Questionnaire (0.00–3.00; no impairment, respective impairment), *LD* lactate dehydrogenase (reference interval 105–333 IU/L), *M* male, *MB* positive muscle biopsy, *MDA5* melanoma differentiation-associated protein 5, *MMT-8* manual muscle testing in 8 muscle groups (0–100 %; muscle strength), *MTX* methotrexate, *MW* muscle weakness, *NA* not available, *PM* polymyositis, *Pred* prednisone, *S* skin rash, *s-CK* serum creatine kinase, *SSA* anti-Sjögren’s syndrome-related antigen A (also called anti-Ro), *TIF1γ* transcription intermediary factor 1-gamma

### Clinical and laboratory assessment

Clinical and laboratory outcome measures were retrieved from the SweMyoNet quality of care register for myositis patients and from medical records. Muscle performance before and after treatment was assessed by the Manual Muscle Test (MMT-8) and the Functional Index-2 (FI-2); ≥15 % increase was defined as improved [17]. The MMT-8 measures isometric muscle strength in eight muscle groups [18] and the FI-2 measures dynamic repetitive muscle performance; it includes seven muscle groups with a maximum of 60 or 120 repetitions for each muscle [19]. Both the MMT-8 and the FI-2 are presented as % of maximal score (100 % = good muscle performance) in Table 1. Serum levels of creatine kinase (CK) and lactate dehydrogenase (LD) were analyzed as routine tests at the Department of Clinical Chemistry, Karolinska University Hospital. Myositis-associated and myositis-specific autoantibodies were tested by RNA immunoprecipitation (IP) and protein IP in Kyoto, Japan, and are presented in Table 1 [20, 21].

### Histopathological and immunohistochemical analyses

Histopathological evaluation of muscle tissue sections was performed by an experienced muscle pathologist on coded sections stained with hematoxylin and eosin. Immunohistochemistry staining was used to identify the presence of inflammatory cells such as T lymphocytes (CD3), macrophages (CD68) and the expression of MHC class I according to a standard protocol [22] using mouse monoclonal anti-CD3 (BD Biosciences, CA, USA), anti-CD68 (Dako Cytomation, Denmark) and anti-MHC-I (My Bio Sourse, CA, USA) antibodies. Isotype-matched irrelevant antibodies were used as negative controls. Conventional microscopic evaluation of the staining was performed and the whole tissue sections were scored for CD3 and CD68 as follows: 0, no positive cells; 1, few positive scattered cells or one infiltrate of inflammatory cells; 2, clusters of positive cells or two infiltrates of inflammatory cells; and 3, several large cellular infiltrates. For MHC-I staining, the sections were scored as follows: 0, no positive fibers; 1, few positive scattered fibers; 2, clusters of positive fibers; and 3, several large areas with positive fibers.

Fiber-type composition was determined by mATPase staining to distinguish between slow-twitch type I and fast-twitch type II muscle fibers [23, 24]. In brief, muscle sections were pre-incubated at acidic or alkaline pH, respectively. Type I fibers emerge in a black color at pH 4.3 in contrast to type II fibers which appears in white; the opposite pattern is observed when pre-incubating at pH 10.3. Semi-quantitative analysis was applied on coded sections for analysis of fiber-type composition; the whole tissue section area was evaluated by counting fibers using a Leica microscope system (BX60; digital camera, Sony CDK-500, Tokyo, Japan). The results are presented as fiber type percentage of the total amount of fibers on the section.

### RNA expression profiling

Expression profiling was performed using Affymetrix Human Genome U133 Plus 2.0 microarrays. Total RNA isolation, cDNA synthesis, cRNA labeling, microarray hybridization, and image acquisition were performed according to the manufacturer’s protocol [25]. The quality control criteria developed at the Children’s National Medical Center Microarray Center for each array were followed [25].

Hybridization signals of the microarrays were recorded using Microarray Suite 5.0 (MAS 5.0) (Affymetrix) and the data were analyzed using GeneSpring 7.0 (Agilent, CA, USA). Genes were filtered with the number of present calls across the 12 arrays analyzed. Genes with at least one present call were selected for statistical analysis using paired *t* test. All profiles have been made publicly accessible via NCBI GEO (http://www.ncbi.nlm.nih.gov/geo/).

Genes with a fold change ≥2 were selected, and a functional analysis of the molecular networks and pathways was performed using the Ingenuity Pathway Analysis (IPA; Ingenuity Systems®, www.ingenuity.com). The significance of the association between the genes in the dataset, biological functions, and pathways was determined by the right-tailed Fischer’s exact test.

### Western blot

Western blot was performed by using a tissue section protocol [26]. The 10-μm muscle sections were lysed in Tissue Protein Extraction Reagent (T-PER; Thermo Scientifics, USA) supplemented with 1× complete protease inhibitor cocktail (Roche Diagnostics GmbH, Mannheim, Germany) and incubated on ice for 30 min. The protein content was determined using a Bio-Rad protein assay (Bio-Rad Laboratories AB, Sweden). Gel electrophoresis was carried out on the NuPAGE® Novex® Bis-Tris gel system (Invitrogen AB, Sweden). Proteins were transferred on a polyvinylidene difluoride membrane using a Trans-Blot SD semi-dry transfer cell (Bio-Rad Laboratories). The membrane was blocked with 5 % milk in phosphate-buffered saline (PBS; 0.1 % Tween-20) and incubated with primary (rabbit polyclonal anti-caspase-1 (Millipore, MA, USA), rabbit polyclonal anti-FKBP5 (Millipore, MA, USA), mouse monoclonal anti-AIM-2 (LifeSpan Biosciences, WA, USA)) (overnight, 4 °C) and secondary (ECL anti-mouse IgG HRP linked (GE Healthcare, UK), ECL anti-rabbit IgG HRP linked (GE Healthcare, UK)) (1 h, room temperature) antibodies. The bands were detected by enhanced chemiluminescence (ECL) and the band intensities were measured using the Gel Doc XR system (Bio-Rad Laboratories). Quantification was performed with normalization against GAPDH as a housekeeping protein.

### Statistical analyses

Clinical and experimental data were analyzed using Wilcoxon signed rank test. The level of significance was set at a *p* value ≤0.05.

## Results

### Effects of treatment on clinical parameters

Clinical data are summarized in Table 1. All untreated patients had a median of 7.5 months (range 0.5–16 months) duration of clinical symptoms to the first biopsy, which was taken as part of the diagnostic work-up. At the time of the second biopsy, after a median of 9 months (range 8–15 months) with immunosuppressive treatment, two out of six adult patients fulfilled the definition of improvement for MMT-8, and four patients improved for FI-2. One out of the six patients achieved the maximum score of 100 % but still had a low test on endurance FI-2, and only one reached the maximum test of FI-2 at the second biopsy, indicating persisting muscle impartment in almost all patients (Table 1). All patients had normal CK values at the second biopsy (Table 1).

### Histopathological and immunohistochemical changes in pre- and post- treatment muscle biopsies

In the pre-treatment biopsy, four patients had detectable inflammatory cells: two had large inflammatory infiltrates, and two had scattered T lymphocytes or macrophages. Five out of the six patients had detectable positive staining for MHC-I expression in muscle fiber membranes, ranging from small areas with discrete staining to large areas with whole fibers expressing MHC-I. In the follow-up biopsy after immunosuppressive treatment, a few scattered T lymphocytes and macrophages were present in one patient, and scattered T lymphocytes were found in another patient. MHC class I expression was expressed in muscle fibers in one of five available follow-up biopsies. In addition, two pre-treatment biopsies showed signs of degenerating or regenerating fibers, but none of the follow-up biopsies showed this.

### Effects of treatment on the overall gene expression

After treatment, the expression of 369 genes was significantly affected (>2.0 fold change) in the muscle tissue of patients, including 126 upregulated and 243 downregulated genes. Gene Ontology analysis demonstrated that the top *Upstream Regulators* statistically relevant for our gene dataset were *Interferon Gamma* (IFNG), *interferon regulatory factor 7* (IRF7), *Interferon type I* (IFNα), *signal transducer and activator of transcription 2* (STAT2) and *Interferon Alfa 2* (IFNA2), which were predicted to be inhibited based on the gene expression changes in the dataset.

### Effects on genes associated with immune response and inflammation

**Fig. 1:**
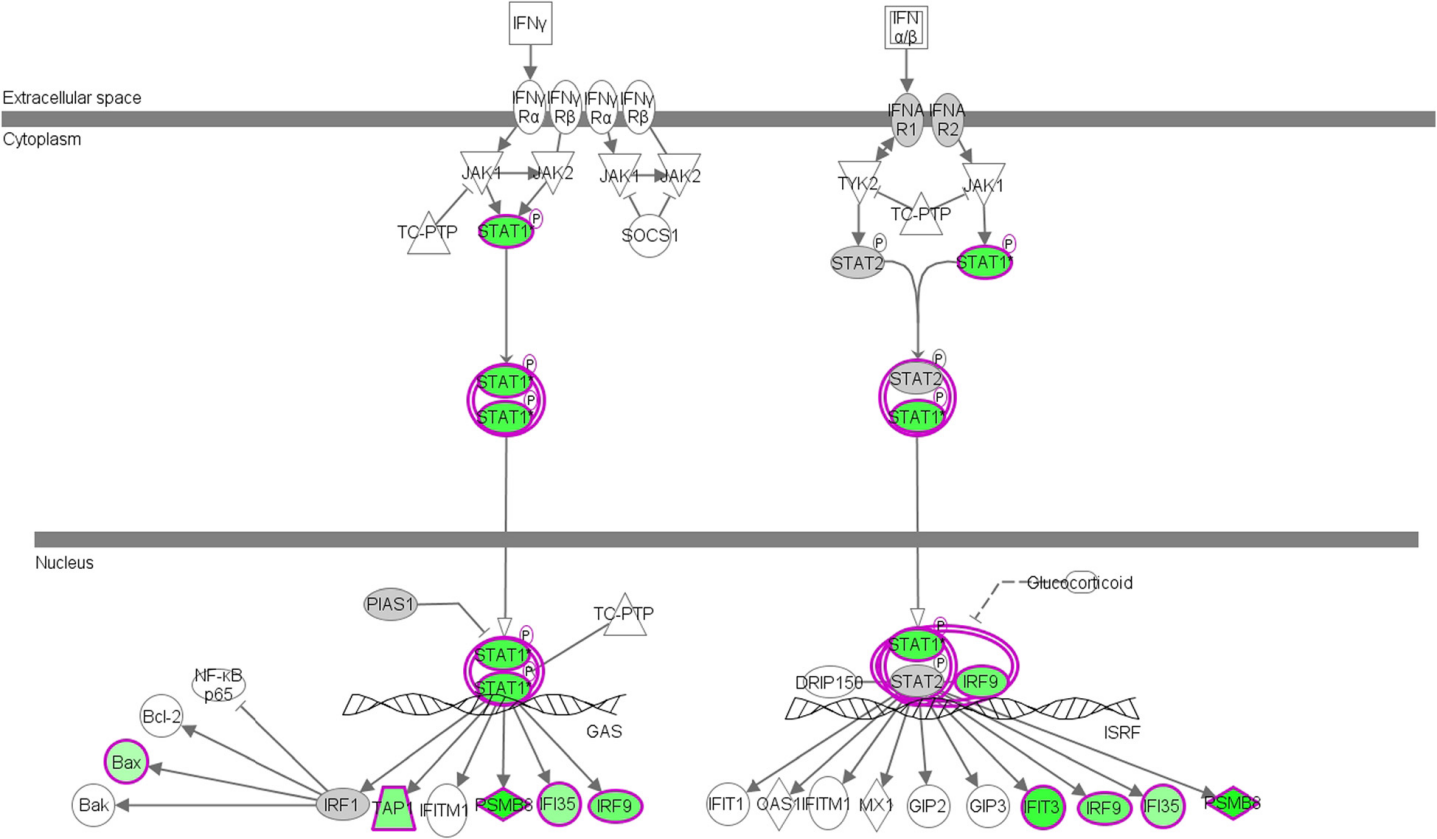
Schematic diagram illustrating the suppression of the interferon (*IFN*) pathway in the skeletal muscle of patients with PM or DM after immunosuppressive treatment determined using the Ingenuity Pathways Analysis knowledge database. *Green* represents significant downregulation of the gene expression, *red* implies significant upregulation, and *grey* specifies changes that did not reach the defined cutoff. A higher intensity of the colors suggests a higher degree of change. No color indicates no presence of this particular gene in our data set

Gene Ontology analysis showed that the expression of 39 out of 43 genes associated with immune response and inflammation was downregulated by treatment (Table 2). Among the downregulated genes, a high representation of HLA-genes encoding *MHC-I* and *MHC-II* (which present antigens to CD8^+^ and CD4^+^ T cells, respectively) was seen. The expression of the co-stimulatory molecules *CD80* and *CD86* was also reduced. Moreover, a variety of chemokine receptors and ligands, both α- and β-chemokines, were downregulated (Table 2). Furthermore, the interferon signaling pathway was strongly downregulated in response to treatment. The expression of 13 genes, which are involved in type I as well as in type II IFN signaling, was reduced (Table 2, Fig. 1). Moreover, *Absent in melanoma 2* (AIM2) and *Caspase-1* (CASP1), components of an inflammasome complex promoting inflammation, were also downregulated. Additionally, specific receptors for pro-inflammatory lipid mediators such as *Prostaglandin E Receptor 4* (PTGER4) and *Cysteinyl Leukotriene Receptor 1* (CYSLTR1) were downregulated by treatment.

**Table 2:** Changes in expression (cutoff 2-fold) of the genes involved in immune responses and inflammation in patients with polymyositis or dermatomyositis after a median of 8.5 months of immunosuppressive treatment

Gene symbol	Gene	Affy #	Fold change	*p*
Immune response and antigen presentation				
CCL2	*chemokine (C-C motif) ligand 2*	216598_s_at	–5.9	0.004
CCL5	*chemokine (C-C motif) ligand 5*	1405_i_at	–3.0	0.043
CCR2	*chemokine (C-C motif) receptor 2*	206978_at	–2.3	0.004
CCR5	*chemokine (C-C motif) receptor 5*	206991_s_at	–2.8	0.027
CD52	*CDW52 antigen (CAMPATH-1 antigen)*	204661_at	–2.7	0.037
CD80	*CD80 antigen (CD28 ag ligand 1, B7-1 ag)*	1554519_at	–2.2	0.034
CD86	*CD86 antigen (CD28 ag ligand 2, B7-2 ag)*	210895_s_at	–2.6	0.013
CHRNA1	*cholinergic receptor, nicotinic, αpolypeptide 1*	206633_at	–2.8	0.028
CNPY3	*trinucleotide repeat containing 5*	1556389_at	–2.1	0.022
CPM	*carboxypeptidase M*	206100_at	2.2	0.028
HLA-DQB1	*MHC class II, DQβ2*	212998_x_at	–2.0	0.033
HLA-A	*major histocompatibility complex, class I, A*	215313_x_at	–2.2	0.012
HLA-G	*HLA-G histocompatibility antigen, class I, G*	211530_x_at	–2.3	0.010
HLA-C	*MHC class I, C*	208812_x_at	–2.2	0.013
HLA-B	*MHC class I, B*	209140_x_at	–2.2	0.017
HLA-F	*MHC class I, F*	204806_x_at	–2.6	0.018
HLA-DQA1	*MHC class II, DQα1*	203290_at	–2.6	0.036
HLA-DQB1	*MHC class II, DQβ1*	209823_x_at	–2.8	0.010
HLA-DPA1	*MHC class II, DPα1*	213537_at	–2.9	0.009
IL-23A	*Interleukin 23, subunit alpha*	217328_at	–5.2	0.005
IL-12RB1	*Interleukin 12 receptor, beta 1*	1552584_at	–2.1	0.020
NMU	*Neuromedin U*	206023_at	2.8	0.028
MMP3	*Matrix metalloproteinase 3*	205828_at	10.7	0.023
IFN pathway				
STAT1	*signal transducer & activator of transcription 1, 91 kDa*	209969_s_at	–3.3	0.008
CXCL10	*chemokine (C-X-C motif) ligand 10*	204533_at	–5.6	0.020
CXCL11	*chemokine (C-X-C motif) ligand 11*	211122_s_at	–5.6	0.015
RTP4	*28kD interferon responsive protein*	219684_at	–5.7	0.028
IRF8	*IFN consensus sequence binding protein 1*	204057_at	–2.4	0.033
ISG20	*IFN stimulated gene 20 kDa*	204698_at	–5.8	0.029
IFI6	*IFNα-inducible protein*	204415_at	–4.7	0.045
IFI30	*IFNγ-inducible protein 30*	201422_at	–2.2	0.036
IFI35	*IFN -induced protein 35*	209417_s_at	–2.6	0.036
IFIT3	*IFN -induced protein w tetratricopeptide repeats 4*	229450_at	–5.1	0.032
IRF9	*IFN -stimulated transcription factor 3, γ*	203882_at	–3.8	0.005
GBP1	*guanylate binding protein 1, IFN-inducible*	202269_x_at	–2.9	0.009
GBP2	*guanylate binding protein 1, IFN-inducible*	242907_at	–2.7	0.005
GBP5	*guanylate binding protein 5*	238581_at	–2.1	0.017
Inflammasome				
AIM2	*absent in melanoma 2*	206513_at	–2.5	0.008
CASP1	*caspase 1, (interleukin 1β convertase)*	211367_s_at	–2.3	0.009
IL18	*interleukin 18 (IFNg-inducing factor)*	206295_at	–2.2	0.042
Eicosanoids				
PTGER3	*prostaglandin E receptor 3 (subtype EP3)*	210832_x_at	3.0	0.013
PTGER4	*prostaglandin E receptor 4 (subtype EP4)*	204897_at	–2.0	0.027
CYSLTR1	*cysteinyl leukotriene receptor 1*	230866_at	–2.8	0.037

### Effects on genes involved in muscle tissue remodeling

A number of genes associated with muscle tissue remodeling were affected by treatment (Table 3). Five genes associated with the ubiquitin-proteasome pathway were downregulated. The GR co-chaperone protein *FK506 binding protein 5* (FKBP5) was upregulated while *Nuclear receptor co-activator 6* (NCOA6) was decreased after treatment. The expression of the genes for *sarcomeric muscle protein α-actinin 3* (ACTN3) and *vinculin* (VCL) was enhanced, suggesting a compensatory increase to cope with the muscle loss due to degeneration. However, the negative regulator of muscle growth *Myostatin* (MSTN) was also upregulated suggesting active inhibition of muscle growth, while *Bone morphologic protein 1* (BMP1) protease that can regulate MSTN by cleaving was downregulated after treatment. Also, *Ras associated with diabetes* (RRAD) and *Myosin binding protein H* (MYBPH) that is involved early in skeletal muscle development was suppressed upon treatment suggesting reduced fiber regeneration. IPA functional analysis based on over-representation and expression direction of genes in our data set predicted the size of muscle cells and development of blood vessels to be reduced after treatment (Z-score –2.108 and –2.509, respectively). These data indicate negative effects of treatment on muscle fiber differentiation and growth. In addition, the expression of the myosin heavy chain 4 (MYH4) and ACTN3, specific markers for fast type II fibers, was upregulated suggesting a fiber type switch towards fast type II fibers in response to treatment.

**Table 3:** Changes in expression (cutoff 2-fold) of genes involved in ubiquitin proteasome pathway, skeletal muscle structure, and remodeling in patients with polymyositis or dermatomyositis after immunosuppressive treatment

Gene symbol	Gene	Affy #	Fold change	*p*
Ubiquitin proteasome pathway				
PSMB8	*proteasome subunit,β type, 8 (large multifunctional protease 7)*	209040_s_at	–6.6	0.003
UBE2L6	*ubiquitin-conjugating enzyme E2L 6*	201649_at	–2.8	0.032
PSMB9	*proteasome (prosome, macropain) subunit, beta type, 9*	204279_at	–2.4	0.005
PSME1	*proteasome) activator subunit 1 (PA28 α)*	200814_at	–2.3	0.007
PSME2	*proteasome activator subunit 2 (PA28β)*	201762_s_at	–2.0	0.012
CNTN3	*ubiquitin-activating enzyme E1C (UBA3 homolog, yeast)*	229831_at	2.4	0.029
Structure proteins and tissue remodeling				
MYBPH	*myosin binding protein H*	206304_at	–6.9	0.036
RRAD	*Ras-related associated with diabetes*	204803_s_at	–3.2	0.013
BMP1	*bone morphogenetic protein 1*	207595_s_at	–2.7	0.001
NCOA6	*Nuclear receptor co-activator*	1568874_at	–3.0	0.041
CACNA1D	*calcium channel, voltage-dependent, L type, alpha 1D subunit*	1555993_at	–2.9	0.035
CHST11	*carbohydrate (chondroitin 4) sulfotransferase 11*	226368_at	–2.1	0.009
MYH4	*myosin, heavy polypeptide 4, skeletal muscle*	208148_at	2.2	0.020
FOXO1	*forkhead box O1A*	202723_s_at	2.3	0.026
MSTN	*growth differentiation factor 8*	207145_at	2.3	0.041
VCL	*vinculin*	200930_s_at	2.4	0.022
TIMP4	*tissue inhibitor of metalloproteinase 4*	206243_at	2.6	0.024
FKBP5	*FK506 binding protein 5*	204560_at	3.4	0.015
ACTN3	*actinin, alpha 3*	206891_at	3.4	0.037

### Effects on genes involved in lipid metabolism

Treatment resulted in significant changes in the expression of genes involved in lipid metabolism (Table 4). Genes responsible for *fatty acid* (FA) uptake and transport such as *fatty acid binding protein 7* (FABP7) and *ATP-binding cassette, sub-family D member 2* (ABCD2) were upregulated. Moreover, genes that promote lipolysis such as *Lipoprotein Lipase* (LPL), *Hormone-sensitive lipase* (LIPE), and *Carboxylesterase 1* (CES1) were also upregulated, while the genes that protect from lipolysis, for instance *Lipid Storage Droplet Protein* (LSDP5), were suppressed suggesting enhanced generation of free FA. Genes associated with FA oxidation and oxidative phosphorylation was not affected (data not shown), suggesting partition of FA into intramuscular lipids. Moreover, genes that favor lipogenesis and lipid storage, e.g. *stearoyl-CoA desaturase* (delta-9) (SCD), *cell death-inducing DFFA-like effector c* (CIDEC), and *ceramide synthase 3* (CERS3), were enhanced (Table 4). In line with these results, based on expression results of genes in the data set, storage of lipids was predicted to be increased (Z-score +2.066). Notably, the expression of *sphingosine kinase 1* (SPHK1) was decreased, suggesting enhanced accumulation of ceramide, an important lipid mediator previously implicated in lipotoxicity [27].

**Table 4:** Changes in expression of the genes involved in lipid metabolism in patients with polymyositis or dermatomyositis after immunosuppressive treatment

Gene symbol	Gene	Affy #	Fold change	*p*
Lipid transport and uptake				
FABP7	*fatty acid binding protein 7, brain*	205029_s_at	10.0	0.002
ABCD2	*ATP-binding cassette, sub-family D member 2*	207583_at	4.55	0.043
APOL6	*apolipoprotein L, 6*	241869_at	–3.12	3.14E-05
Lipid accumulation and lipolysis				
SCD	*stearoyl-CoA desaturase (delta-9)*	223839_s_at	3.86	0.042
CIDEC	*cell death-inducing DFFA-like effector c*	219398_at	3.53	0.049
CERS3	*ceramide synthase 3*	1554252_a_at	3.2	0.021
CES1	*carboxylesterase 1*	209616_s_at	2.98	0.028
MSTN	*myostatin*	207145_at	2.28	0.041
CNR1	*Human CB1 cannabinoid receptor*	213436_at	2.14	0.009
LPL	*lipoprotein lipase*	203549_s_at	2.05	0.022
LIPE	*lipase, hormone-sensitive*	213855_s_at	2.00	0.034
ACSL3	*fatty-acid-Coenzyme A ligase, long-chain 3*	236168_at	–3.87	0.048
LSDP5	*Lipid Storage Droplet Protein 5*	1560457_x_at	–2.61	0.030
SPHK1	*sphingosine kinase 1*	219257_s_at	–2.20	0.037

### Confirmation of changes at the protein level

**Fig. 2:**
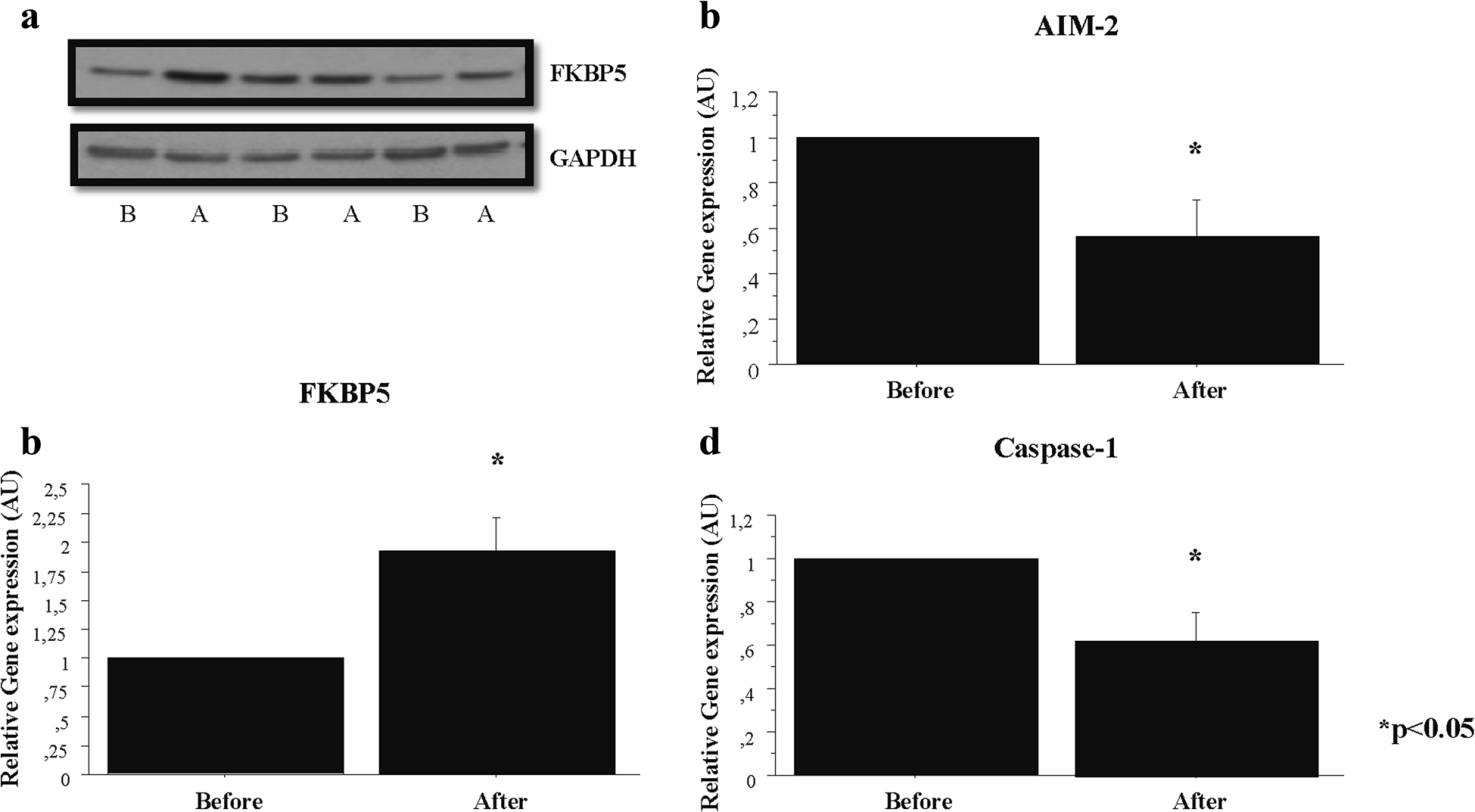
FKBP5 was significantly upregulated after glucocorticoid treatment. AIM-2 and Caspase-1 expression was significantly downregulated after glucocorticoid treatment. **a** The expression of FKBP5 and GAPDH before (*B*) and after (*A*) glucocorticoid treatment was determined by Western blot. **b** Densitometry plots showing FKBP5 expression normalized to GAPDH and expressed as fold-increase relative to before sample. **c** Densitometry plots showing AIM-2 expression normalized to GAPDH. **d** Densitometry plots showing Caspase-1 expression normalized to GAPDH. *p < 0.05

To confirm changes in gene expression at the protein level by Western blot we selected eight genes that were significantly changed. Two of the chosen genes are involved in the inflammatory pathway (AIM-2 and Caspase-1), which were both downregulated after treatment. The FKBP5 gene is implicated in muscle tissue remodeling and was upregulated after treatment. Using Western blot, we confirmed significantly reduced protein expression of AIM-2 and Caspase-1 (*p* < 0.05), suggesting a reduction in inflammatory signaling (Fig. 2). We also observed an increased protein expression of FKBP5 (*p* < 0.05), supporting a negative effect on muscle tissue remodeling by the immunosuppressive treatment. No significant changes for EP3, EP4, CystLTR1, FOXO1A, and FABP7 were detected at the protein level (data not shown).

### Effects on fiber type composition

**Fig. 3:**
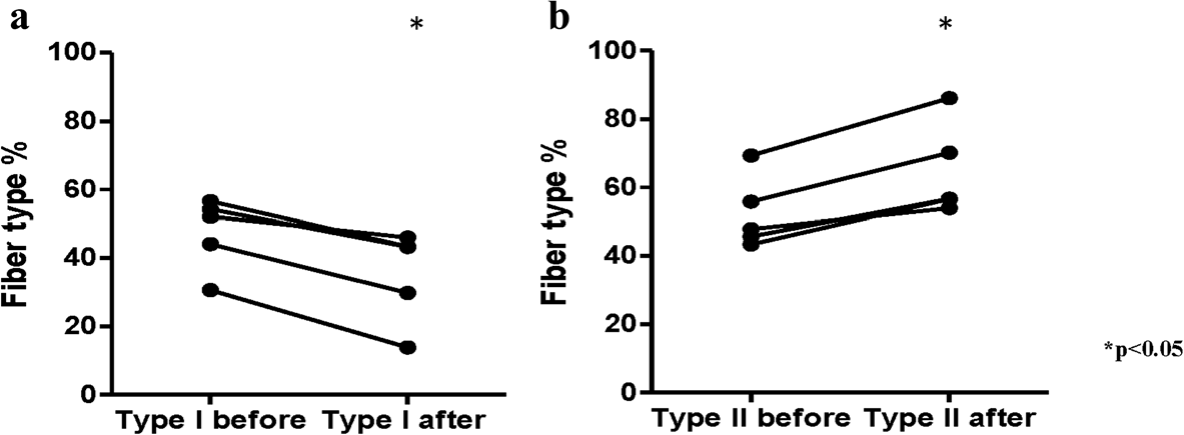
The fiber type composition was significantly different after glucocorticoid treatment. **a** The percentage of type I fibers was significantly decreased after glucocorticoid treatment from median 52 % to median 43 % (*p < 0.05). **b** The proportion of type II fibers was significantly increased after treatment from median 48 % to median 57 % (*p < 0.05)

A switch in fiber types was seen in the post-treatment biopsy as compared to that before treatment. The percentage of type I fibers had decreased significantly after treatment, from a median of 52 % (range 31–57 %) to 43 % (range 14–46 %) (*p* < 0.05). In contrast, the proportion of type II fibers was significantly higher after treatment (before treatment, median 48 % (range 43–69 %); after treatment, 57 % (54–86 %); *p* < 0.05), thus confirming the gene expression data (Fig. 3).

## Discussion

In the present study, in which adult patients improved but none had recovered muscle strength at the follow-up biopsy, we found that immunosuppressive treatment of newly diagnosed PM and DM patients had suppressive effects on gene expression of immune and inflammatory pathways, including type 1 IFN and inflammasome pathways, in skeletal muscle. However, we also observed changed expression of genes involved in skeletal muscle tissue remodeling indicating protein breakdown and reduced muscle regeneration, which may negatively affect muscle regeneration and growth. Furthermore, we found altered expression of genes associated with lipid uptake, lipolysis, and lipid accumulation in response to treatment, indicating complex effects on intramuscular lipid metabolism that may also have a negative effect on muscle performance. Among the immune and inflammatory pathways suppressed by treatment, the downregulation of type I IFN pathways in muscle tissue was most striking. It is well recognized that the type I IFN pathway is activated in patients with autoimmune diseases including IIM [28, 29]. A significant upregulation of IFN-inducible genes in muscle biopsies from PM and DM patients was detected compared to age-/sex-matched controls [30, 31]. The high overexpression of interferon-inducible genes was also demonstrated in whole blood from both PM and DM patients [32]. Moreover, a recent study of peripheral blood gene expression has revealed that IIM patients displayed a predominant IFNα-mediated response program [29]. The expression of type I IFN-inducible genes in whole blood correlated with disease activity in PM and DM patients and was reduced after immunomodulatory therapies [32, 33]. Our novel finding that immunosuppressive treatment suppressed the IFN pathway in muscle tissue from PM and DM patients is in agreement with these previous reports. Our results provide additional evidence supporting the beneficial effects of conventional immunosuppressive treatment in myositis, through inhibition of the IFN pathway and reduced formation of pro-inflammatory mediators in muscle tissue.

Another finding was downregulation of genes involved in inflammasome activity in response to treatment, which was confirmed at the protein level for AIM-2 and Caspase-1. Our findings have added insights into the favorable effects of conventional immunosuppressive treatment, which includes inhibition of the inflammasome pathway in muscle tissue in patients with PM or DM, as well as several other pathways associated with immune response and inflammation, which was validated by immunohistochemistry confirming a low degree of inflammation in the post-treatment biopsies as assessed by CD3, CD68, and MHC-I expression.

However, our group has previously demonstrated an insufficient effect of immunosuppressive treatment on PGE_2_ and LTB_4_ pathways associated with the persistent expression of mPGES-1, COX-1, and 5-LO proteins in myositis muscle despite treatment [13, 14]. In line with these observations, we did not detect any alterations in the gene expression of these enzymes or changes at the protein level for the eicosanoid receptors *EP3*, *EP4*, and *CysLTR1*. The receptors were expressed at the protein level in muscle from patients with myositis before and after treatment, suggesting that PGE_2_ and LT might contribute to chronic inflammation and muscle wasting and these pathways could be potential targets for new therapies.

Importantly we found signs in the gene expression profiles after treatment indicating an effect on muscle remodeling. We observed downregulation of several genes in the ubiquitin-proteasome pathway and also increased expression of structural proteins such as α-actinin and vinculin, indicating an increase in muscle mass. Reversely, we detected increased expression of myostatin, suggesting inhibition of myogenesis and a negative effect on muscle growth. Furthermore, downregulation of RRAD and MYBPH could also be a sign of reduced muscle regeneration. RRAD expression was elevated during skeletal muscle development as well as in adult muscle post-injury [34]. FKBP5 is an essential functional regulator of the GR complex and is associated with muscle tissue alteration; it plays an important role in basic cellular processes and in immunoregulation involving protein folding and trafficking [35]. We observed an increased protein expression of FKBP5, implicating a negative effect on muscle tissue remodeling. Overall, these data point to negative effects of conventional immunosuppressive treatment on muscle regeneration and growth. Furthermore, the enhanced gene expression of specific markers for fast type II fibers, MYH4 and ACTN3, suggest a fiber-type switching towards the type II fibers in response to treatment, which was confirmed by analysis of fiber-type composition. This observation is in agreement with the clinical problem of low muscle endurance as measured by FI-2 and with previous data reporting a shift towards fast twitch type II fibers in patients with chronic PM or DM which interestingly could be reversed by exercise [36, 37].

A third pathway that we found to be altered in muscle tissue after immunosuppressive treatment relates to lipid metabolism. The balance between lipid production and oxidation is essential for normal cell functions; thus, an excess of FFA is converted to triacylglycerol for intracellular lipid storage. The dysregulation of this process leads to the production of lipotoxic lipid intermediates (ceramides, diacylglycerol, fatty acyl CoA) that might cause cell dysfunction or death [38]. A novel observation from our study is that immunosuppressive treatment including GC might affect lipid storage in skeletal muscle. In addition, upregulated CERS3 suggests an enhanced accumulation of ceramide which has previously been linked to insulin resistance [39]. Moreover, ceramide has been implicated in skeletal muscle dysfunction and fatigue in chronic diseases and in mouse muscle fibers in vitro [40, 41]. Additional detailed studies are needed to define lipid profiles in muscle tissue from myositis patients in comparison with healthy individuals and in relation to immunosuppressive treatment. Notably, patients with juvenile DM are at risk of developing lipodystrophy, associated with loss or redistribution of subcutaneous fat [42]. The lipodystrophy is accompanied by metabolic abnormalities such as insulin resistance, diabetes and dyslipidemia, and may occur as a result of inflammation. Our study included adult patients, although there is very little known about lipodystrophy in adult patients with PM or DM. There is a case study from 2007 describing a woman suffering from a typical DM which developed lipodystrophy and insulin resistance [43]. Although worth mentioning, there is no evidence that standard therapies for DM causes lipodystrophy.

A strength of our study is the paired muscle biopsy samples, with two biopsies taken from the same individuals and the repeated biopsy that was taken regardless of clinical signs of a flare. A paired sample study design reduces the problem of inter-individual variations. Nevertheless, our current study has several limitations: one of them is the low number of patients included and the heterogeneity in diagnoses of PM and DM and in the degree of histopathological changes before treatment. Also, no magnetic resonance imaging (MRI) was performed before the biopsies were taken which could have enhanced the detection of inflammation in the muscle. Differences in typical histopathological features in muscle biopsies seen in PM and DM suggest that different mechanisms may contribute to the muscle inflammation. However, several studies on cytokine and chemokine expression have not revealed significant differences between PM and DM, suggesting that inflammatory molecular pathways may be shared. One patient with typical DM features and muscle weakness had no signs of MHC class expression on muscle fibers, which could be explained by the sometimes patchy distribution of MHC class I expression. Another limitation is the inconsistency in the immunosuppressive treatment used in combination with GC, as it was given based on the decision of the treating physician, although all patients were treated with high doses of GC. Furthermore, the total expected duration of immunosuppressive treatment in patients with PM or DM is often 2–3 years. Here, we chose to take a repeated biopsy after approximately 9 months, which is not likely to show the final repaired muscle but rather an effect of the immunosuppressive treatment on molecular pathways (which was the aim of our study). Despite the heterogeneity in diagnosis and treatment and the low degree of inflammatory cell infiltrates in two patients before treatment, we could still see significant downregulation of genes involved in inflammation, supporting the beneficial effect of the immunosuppressive treatment on the inflammatory pathway. One patient developed type 2 diabetes after the start of immunosuppressive treatment. None of the other patients had medications or conditions that could impact muscle metabolism. Furthermore, details on diet were not included. In recent years, our group has shown that intensive exercise can have a positive influence on muscle health [44]. Four out of the six patients in the present study did exercise regularly, which might have counteracted some of the damage induced by the oral corticosteroid treatment. Moreover, it is not possible to distinguish between the relative contribution of the disease progress and the immunosuppressive treatment on the outcome in this study. To address this question an experimental model should be considered. Due to the limited number of patients the results need to be interpreted with some caution and need to be replicated in a larger cohort of patients.

## Conclusions

In conclusion, a majority of genes involved in immune response were downregulated in muscle tissue from patients with PM or DM after conventional immunosuppressive treatment. In addition, genes involved in protein degradation and muscle regeneration were altered, indicating insufficient muscle tissue remodeling, and, finally, the expression of genes related to lipid metabolism was affected by treatment, suggesting intramuscular lipid accumulation leading to skeletal muscle dysfunction. These findings provide new plausible explanations for the persistent muscle weakness and fatigue observed in patients despite treatment, and diminished tissue inflammation, and at least some of these may be affected in a beneficial way by combining immunosuppressive treatment with physical exercise.

## Abbreviations

ABCD2, ATP-binding cassette, sub-family D member 2; ACTN3, sarcomeric muscle protein α-actinin 3; AIM2, Absent In melanoma 2; BMP1, Bone morphologic protein 1; CASP1, Caspase-1; CD3, T lymphocytes; CD68, macrophages; CERS3, ceramide synthase 3; CES1, Carboxylesterase 1; CIDEC, cell death-inducing DFFA-like effector c; CK, creatine kinase; CYSLTR1, Cysteinyl Leukotriene Receptor 1; DM, dermatomyositis; ECL, enhanced chemiluminescence; FA, fatty acid; FABP7, fatty acid binding protein 7; FI-2, Functional Index-2; FKBP5, FK506 binding protein 5; GC, glucocorticoids; GR, glucocorticoid receptor; GRE, glucocorticoid response element; HAQ, Health Assessment Questionnaire; IFNA2, interferon alfa 2; IFNG, interferon gamma; IFN, interferon; IFNα, interferon type I; IIM, idiopathic inflammatory myopathies; IL, interleukin; IPA, Ingenuity Pathway Analysis; IRF7, interferon regulatory factor 7; LIPE, Hormone-sensitive lipase; LPL, Lipoprotein Lipase; LSDP5, Lipid Storage Droplet Protein; LTB_4_, leukotriene B_4_; MHC, major histocompatibility complex; MMT-8, Manual Muscle Test; MRI, magnetic resonance imaging; MSTN, Myostatin; MYBPH, Myosin binding protein H; MYH4, myosin heavy chain 4; NCOA6, nuclear receptor co-activator 6; PGE_2_, prostaglandin E_2_; PM, polymyositis; PTGER4, prostaglandin E Receptor 4; RRAD, Ras associated with diabetes; SCD, stearoyl-CoA desaturase; SPHK1, sphingosine kinase 1; STAT2, signal transducer and activator of transcription 2; TNF, tumor necrosis factor; VCL, vinculin
